# Performance for Fly Ash Reinforced HDPE Composites over the Ageing of Material Components

**DOI:** 10.3390/polym14142913

**Published:** 2022-07-18

**Authors:** Mohammed N. Alghamdi

**Affiliations:** Department of Mechanical Engineering Technology, Yanbu Industrial College, Yanbu Al-Sinaiyah City 41912, Saudi Arabia; alghamdim@rcyci.edu.sa

**Keywords:** fly ash, HDPE, polymer composite, environmental ageing, mechanical properties

## Abstract

The by-product abundances of fly ash allow them to be used as the reinforcing filler for high-volume and high-performance thermoplastic composites. However, the durability of the composites remains questioned as polymer degradation during environmental weathering creates brittle materials, leading to surface cracks, which potentially release hazardous fly ash particles into the environment. This paper reports the effect of environmental ageing (UV and moisture exposure) on the morphological and mechanical properties of fly ash mixed high-density polyethylene (FA/HDPE) composites with three dissimilar weight fractions (5, 10 and 15 wt%) of filler and compared the results with similarly aged neat HDPE samples. The consequence of environmental ageing on the elevated mechanical properties of composites is investigated. Fifteen wt% fly ash reinforced composite appears to have better morphological and mechanical properties after 20 weeks of ageing, with only ~5 and ~9% reduction in Young’s modulus and tensile strength, respectively. The driving factors controlling the ageing effects are broadly discussed and recommendations are made for research advancements.

## 1. Introduction

Polymer matrix composites that incorporate reinforcing particles [1,2,3], fibres [4,5,6,7] or fabrics [8,9,10] are of superior stability, and physical properties show enhanced robustness of the composite systems. Petroleum-derived thermoplastics are amongst the best choices for matrix materials, and the selection of reinforcing elements include but are not limited to carbonous particles or a mixture of such kinds [2,11,12,13], non-carbonous particles [14,15,16,17], carbon fibre, glass fibres and cellulose fibres. Nanoparticles, such as carbon nanotubes [18,19] and graphene [20,21,22] reinforced composites have also attracted a high degree of interest. However, the widespread uses of these nanocomposites for engineering applications and frequent use may create unintentional pollution of land or water due to nanoparticle release over time, increasing the risks of exposure to humans [23,24,25]. Many studies on the ageing of diverse nanocomposites have shown that there exists a significant release of reinforcing nano fillers resulting from environmental ageing [26,27]. Considering this issue with nano scale fillers, micro scale particles, such as fly ash reinforcement, could bring less impact to the environment.

Fly ashes are abundant material usually generated as by-product in different types of heavy manufacturing facilities such as cement, oil, and coal-driven power plants [28,29]. Disposal of fly ash is a great concern and it is highly expected that fly ash can be used to produce commercially valuable products [30,31]. For instance, incorporation of fly Ash with construction materials can offer high quality and economical products such as pre-mixed materials for construction sites [32,33].There have been significant research advances reported on the ongoing work of fly ash-based geopolymer concrete of high interfacial strength [34,35,36], providing the practical feasibility of using fly ashes in heady construction industries. Some studies also reported the effective dosage rate of additivewithin a composite developed for construction application [37]. In regard to the fly ash filler, the concentration should be under a specified threshold in order to eliminate the risk of abrasion-related microparticle release [38]. Following the impression of combining cementitious materials with fly ash, many researchers have extended the reinforcing technology beyond construction industries, including polymer composites [39,40,41]. Scientists also found high interfacial compatibility between fly ash and plastics, making them suitable for fabricating composites of such kind [40,42,43]. The ceramic nature of fly ash particles improves the mechanical performance and physical properties of their reinforced composites, allowing them to be used in several heavy industries, including automotive, aerospace and construction. Choice of plastics includes but not limited to polypropylene (PP), polyethylene, and polyamides (PA). Among these thermoplastic polymers, high-density polyethylene (HDPE) offers high stability and is used in several application fields, including automotive components, packaging of consumables, building materials and electronics [44,45]. HDPE is classified a semicrystalline plastic that is mechanically strong, and resistant to moisture and chemical compounds [46]. These features are particularly beneficial to resist the environmental ageing of the polymers and to prevent the release of microplastics. In regard to the composites of HDPE, it is highly important to investigate such stability features as a multi-component material is not similarly susceptible to environmental ageing as that happens in a single-component polymeric material.

Environmental ageing(e.g., UV or moisture exposure) modifies the mechanical, physical and morphological properties of polymers [47]. Under prolonged UV exposure, polymers undergo chemical cross-linking hence photo degradation, providing inferior mechanical properties of such [4]. Several studies have shown that the use of carbonaceous fillers enhances thermal stability in polymer composites with the cost of material degradation and potential release of the fillers [48,49,50]. Accelerated weathering tests are typically used to examine the variations of material properties against the extent of ageing. This test simulates weather conditions, such as dry and wet environments, and estimates the potential long-term durability of the subject material. Physical, mechanical or morphological characterisation of the subject material can indicate the extent of degradation and the release of reinforcing fillers due to the environmental ageing. While investigation of the stability and degradation of plastic nanocomposites by environmental ageing for understanding the risk of released nano- and micro-scale additives has been performed by a few researchers [51,52,53,54], there has been no such study performed for plastic composites with fly ash fillers. This paper reports the effect of environmental ageing (UV and moisture exposure) on the mechanical and morphological properties of fly ash reinforced HDPE composites considering three different weight fractions (5%, 10% and 15%) of filler and compared the results with similarly aged neat HDPE samples. Loading of fly ash fillers would provide better mechanical properties to the composites and our aim is to investigate how the environmental ageing would affect the elevated mechanical properties. We anticipate that fly ash can act as a stabiliser with respect to degradation of HDPE composite subjected to UV light and high moisture conditions. It is obvious that the concentration of filler fly ash determines the extent of resistance to degradation of this plastic composite. The driving factors behind the ageing effects are comprehensively discussed, and a few recommendations are made for further improvement.

## 2. Materials and Methods

### 2.1. Materials and Compositions

Petroleum-derived thermoplastic HDPE was sourced from Saudi Basic Industries Corporation (SABIC HDPE P6006N), Riyadh, Saudi Arabia. This HDPE material is suitable for compounding into structural components without being much affected in prolonged natural weathering conditions. Fly ash samples (particle size ranging 50–90 µm) were supplied by MARAFIQ power plant, Yanbu, Saudi Arabia. The carbon content of the as-received sample is in the range of ~89–92 wt% [55]. Some metallic micro-particles are also present in the sample, including but not limited to aluminium (~0.0003 wt%), calcium (~0.0005 wt%), magnesium (~0.007 wt%), iron (~0.007%), sodium (~0.001 wt%), nickel (~0.001 wt%) and vanadium (~0.009 wt%). Insignificant amounts of chromium, copper, lead, zinc, and barium are also present in the sample [55].

### 2.2. Experimental Methods

#### 2.2.1. Composite Sample Preparation

The conventional process of melt compounding was used in this study, followed by injection moulding to fabricate samples for tensile testing. Five, 10 and 15 wt% fly ash samples were rotary mixed with as-received HDPE granules and melt compounded using a HAAKE PolyLab Mixer (Thermo Fisher Scientific Inc., Waltham, MA, USA) at 220 °C for 30 min. Then, well-mixed composite batches were removed from the compounder and cooled at normal environment. We name the batches FA5%/HDPE (contains 5 wt% fly ash), FA10%/HDPE (contains 10 wt% fly ash) and FA15%/HDPE (contains 15 wt% fly ash). All the batches of fly ash mixed HDPE (FA/HDPE) compounds and a pure HDPE sample were injection moulded using HAAKE MiniJet II (Thermo Fisher Scientific Inc., Waltham, MA, USA) at 240 °C for 10 sec, forming dumbbell-shaped samples for tensile testing.

#### 2.2.2. Thermogravimetric Analysis

A portion of each final sample was used to perform thermogravimetric analysis (TGA) to determine the processing temperature, degradation pattern and actual fly ash content present in the final samples. In the TGA chamber, neat HDPE and FA/HDPE materials were heated from 25 to 800 °C at a heating rate of 5 °C/min. The mass of each sample was approximately 10 mg. The tests were performed in air (with a purge rate of 20 mL/min) so that the environmental condition could be replicated during melt processing and relevant degradation of the polymer.

#### 2.2.3. Ageing Tests

Ageing tests were performed in an accelerated weathering chamber (QUV accelerated weathering tester, Q-LAB, Westlake, OH, USA) under ultraviolet A (UV-A) bulbs with a wavelength of 400 nm. This wavelength is suitable for ageing experiments rather than burning due to long penetration depth of the radiation. The samples were exposed in the active chamber for 20 weeks, with cycles being: (1) UV-A: 1.55 W/m^2^/nm, 60 °C, 8 h, and (2) Condensation: 50 °C, 4 h.

#### 2.2.4. Characterization Techniques

Scanning electron microscopy (SEM) was initially used to examine the surface degradation of aged samples. Careful consideration was also taken to observe the extent of fly ash particles exposed due to prolonged UV irradiation. Further, fracture surface of the sample interiors was exposed by cryogenically (at −196 °C) breaking them in liquid nitrogen and taken for SEM studies. This observation indicates the extent of filler-matrix adhesion within the composite and gives a measure of interfacial shear strength (IFSS). Since IFSS directly contributes to the mechanical properties of the composite system, tensile tests were conducted for all the aged materials to understand their durability and stability over the ageing period. Multiple test samples were used for ageing tests, and one of each sample type was taken out every five weeks and characterised to understand the overall degradation pattern.

## 3. Results and Discussion

### 3.1. Thermogravimetric Properties

There is a possibility that the melt compounding process can result in the loss of polymer fraction of different batches, being attached with the hot internal wall of the mixer. Thermogravimetric tests were carried out for newly moulded neat HDPE and FA/HDPE samples to study their degradation behaviour, apparently to confirm the presence of expected fly ash content in all the samples of interest. The uniformity of filler concentration is crucial when the composite is exposed to UV light and weathering over a prolonged period of time. It is anticipated that fly ash fillers will absorb light from the whole range of the source spectrum, protecting HDPE from much adsorption of high energy photons. The resistance to UV radiation is also subject to the scattering of the photon adsorbing fillers in the base matrix and relevant concentration.

**Figure 1:**
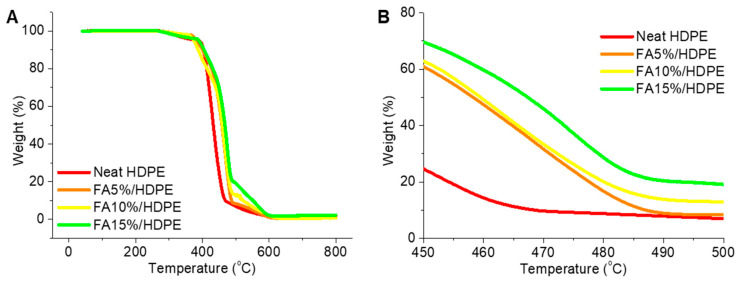
Thermogravimetric test results of newly moulded neat HDPE and FA/HDPE composite materials: (**A**) general trend of degradation, (**B**) understanding of different sample degradation pattern at ~500 °C to evaluate the filler concentration and uniformity.

Figure 1 shows the TGA test results obtained from neat HDPE and FA/HDPE materials. In Figure 1A, all the samples started to lose weight at ~280 °C and fully degraded at ~600 °C. Thermogravimetric pattern can be understood better at ~500 °C (Figure 1B), where neat HDPE and FA/HDPE samples show different extents of material degradation. The composite samples show gradually higher thermal stability as the amount of filler increases. The main driving factor behind such behaviour is the high thermal stability of metal microparticles in bulk fly ash samples. At ~500 °C, the difference between weight degradation is ~5%, which confirms the presence of 5–15 wt% fly ash fillers in a 5 wt% increment. The filler uniformity will also play a crucial role in the mechanical properties of the UV irradiated composites, fly ash particles providing a consistent stabilising effect against light and other weather conditions.

### 3.2. Surface Morphology

HDPE consists of a semicrystalline structure with spheroidic bunches of crystals enclosed by a continuous amorphous phase. During the ageing process in QUV chamber, the elasticity of both neat HDPE and FA/HDPE samples is meant to be decreased. UV exposure and condensation cycles could result in photo oxidation and hydrolysis, producing environmental stress cracking. In this regard, diffusion of oxygen through the amorphous region occurs frequently, but diffusion through the crystalline regions is fairly restricted. Consequently, the majority of environmental ageing happens at the spherulite boundaries, weakening the effective adhesion that holds the crystalline regions together.

**Figure 2:**
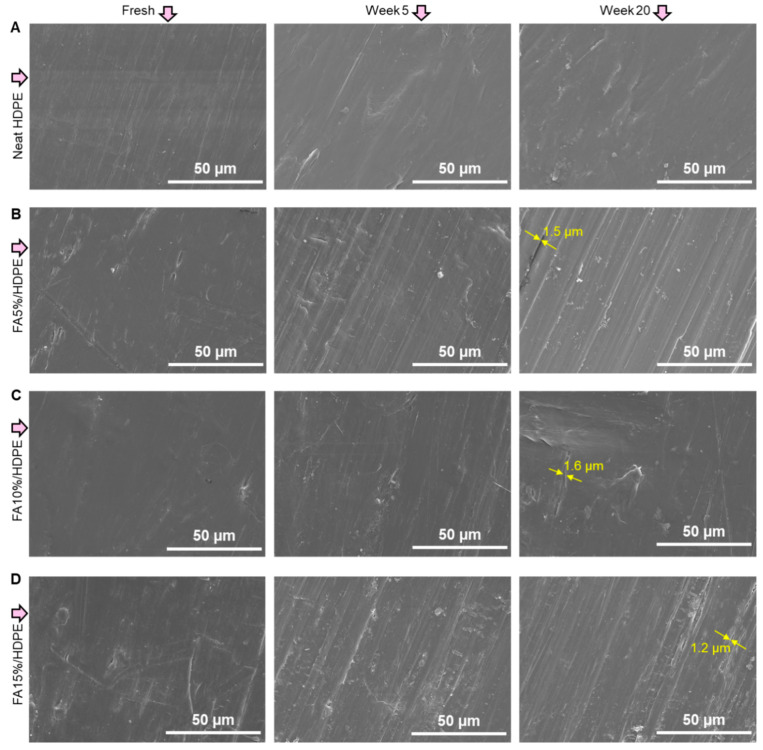
Surface morphology of materials before and after ageing in a QUV chamber: (**A**) neat HDPE, (**B**) FA5%/HDPE, (**C**) FA10%/HDPE, (**D**) FA15%/HDPE. (Arrows point to the minimum cracks initiated after 20 weeks of ageing).

We here demonstrate the extent of surface cracks that might be produced after the ageing tests of neat HDPE and FA/HDPE samples. Figure 2 shows SEM images of freshly made and aged (5 and 20 weeks) samples. While HDPE is inherently a weather-resistive material, it is expected that the FA/HDPE samples will also show similar surface morphology after ageing. The SEM images are therefore evidential, showing very little extent of wear and cracks on the surface of FA/HDPE samples. While our ageing study on FA/HDPE composites is new in its kind, the significance of less weathering effect can be understood from past ageing studies with other particle reinforced HDPE composites. For example, the extent of ageing-related cracks (crack widths <2 µm after 20 weeks of ageing) in our composites are insignificant compared to the previous work on carbon black and titanium dioxide incorporated wood-HDPE composites [56]. This is promising as our ultimate aim is to investigate the reservedness of elevated mechanical properties of FA/HDPE composites after the environmental ageing. It is worth noting that the QUV chamber was run for 20 weeks, and further prolonged UV exposure could lead crack propagation that usually increases over time and breaches the layer adjacent to the surface.

### 3.3. Filler-Matrix Morphology 

Prolonged ageing could change the macro- and micromolecular structure of HDPE and could also trigger the deterioration of chemical and physical properties. The amount of oxygen diffusion is substantial during an accelerated ageing test, particularly affecting the rapidly oxidising polymers and their compounds. The semicrystalline nature of HDPE materials allows them to oxidise entirely in the amorphous portion as the crystal phase is highly resistant to oxygen diffusion. One way to understand the extent of degradation is to study the interior of materials.

**Figure 3:**
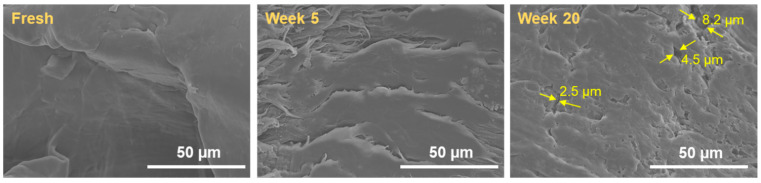
Fracture surface morphology of a neat HDPE sample before and after ageing in a QUV chamber. (Arrows point to the minimum cracks propagated into the polymer interior after 20 weeks of ageing).

In this work, neat HDPE and FA/HDPE samples were cryogenically broken using liquid nitrogen and the fractured surfaces were investigated for material interior degradation. Cryo-fracturing in liquid nitrogen achieved extremely low temperatures under strong thermally induced stresses, and the fracture occurs without damaging the actual structure of materials. Figure 3 shows the SEM images of the fracture surface of neat HDPE samples before and after ageing. The initiation and propagation of the cracks continued as ageing progressed, and the HDPE test sample became significantly brittle after prolonged ageing. It is evident that significant polymer degradation occurred (with >8 µm of crack width produced within the sample) after 20 weeks of ageing, and further UV exposure would result in premature failure of the material.

**Figure 4:**
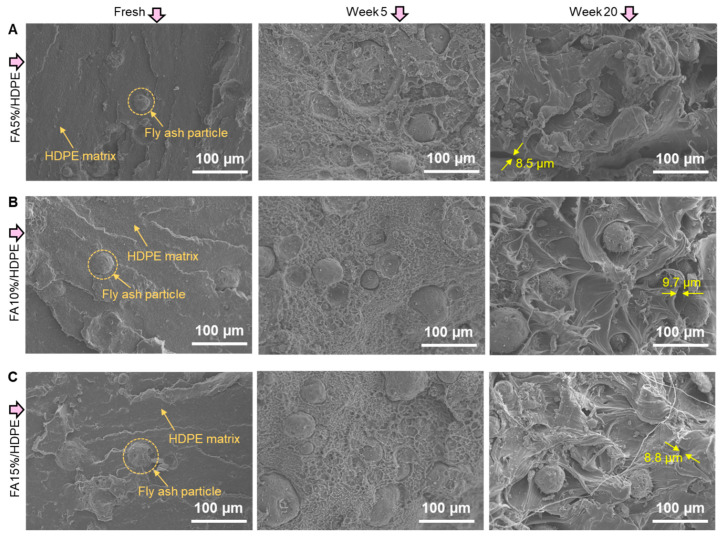
Fracture surface morphology of FA/HDPE samples before and after ageing in a QUV chamber: (**A**) FA5%/HDPE, (**B**) FA10%/HDPE, (**C**) FA15%/HDPE.

Figure 4 shows the cryogenically fractured surfaces of FA/HDPE composites. While studying the surface of aged polymer composite is prospective to the wear or crack initiation, understanding the filler-matrix interfacial morphology is crucial for the performance, i.e., mechanical properties. The filler-matrix interface of differently aged composites is different, with the texture being similar for HDPE at similar ageing times. The extent of polymer degradation is logically identical as all the samples contain the same grade of HDPE precursor. Several cracks are noticed in the HDPE matrix phase after 20 weeks of composite ageing. The pores with random sizes are apparent, with the largest size range being ~8–10 µm. However, as in other polymer composites, filler matrix adhesion will be the driving factor in increased mechanical properties, compensating the loss of properties due to the matrix degradation. Although the fly ash concentration is different in different FA/HDPE samples, their interfacial adhesion with the surrounding polymer matrix remains strong. As can be seen in the SEM images of 20 weeks aged samples, cryogenic fracture caused the exposure of fly ash particles, but the pull out comprises a random layer of HDPE polymer on the particle surface. This phenomenon is attributed to high IFSS between the filler-matrix interfaces and contributes directly to the superior mechanical properties of bulk composites.

### 3.4. Mechanical Properties

**Figure 5:**
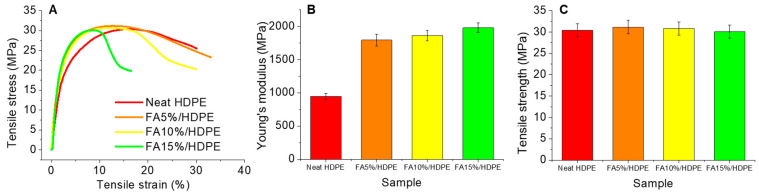
Tensile properties of neat HDPE and FA/HDPE samples: (**A**) representative stress-strain curves, (**B**) comparative Young’s modulus, (**C**) comparative tensile strength.

The tensile behaviour of neat HDPE and 5–15 wt% fly ash reinforced HDPE composites is shown in Figure 5. Figure 5A shows the representative tensile stress vs. tensile strain curves, showing a gradual decrease in tensile strain with increasing fly ash content. This phenomenon is related to the presence of high interfacial bonding, i.e., IFSS between rigid fly ash particles and the surrounding HDPE matrix phase. Figure 5B shows that the incorporation of fly ash gives higher Young’s modulus to their reinforcing HDPE composites, with higher filler concentration providing a higher modulus value. For instance, a FA15%/HDPE composite sample showed Young’s modulus of ~1930 MPa, which is a ~200% increase compared that of a neat HDPE sample. While the enhancement of modulus is expected, it is also crucial that the composite sample at least preserve their tensile strength value, not being lower than a neat HDPE material. Compared to neat HDPE, while a slight increase in the tensile strength was noticed for FA5%/HDPE, a further increase in the fly ash concentration lowered the corresponding tensile strengths. However, measured tensile strengths were either similar or slightly higher compared to neat HDPE, aligning with our previous anticipation of preserved strengths.

**Figure 6:**
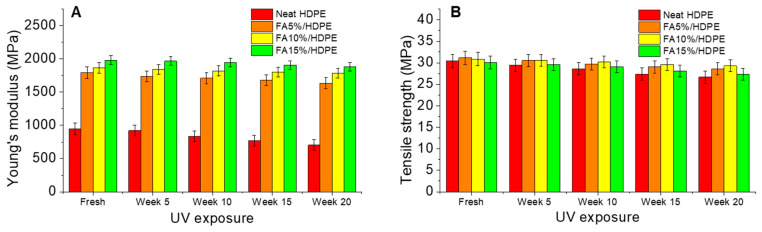
Tensile properties of neat HDPE and FA/HDPE samples evaluated before and after a few environmental ageing periods: (**A**) comparative Young’s modulus, (**B**) comparative tensile strength.

The intensity of environmental ageing and ageing duration has critical effects on the tensile properties of the polymers and their composites. As shown in Figure 6, the tensile properties of both neat HDPE and FA/HDPE materials gradually deteriorate as the ageing duration increases from 5 weeks to 20 weeks under ageing cycles of (1) UV-A: 1.55 W/m2/nm, 60 °C, 8 h and (2) Condensation: 50 °C, 4 h.

Figure 6A shows Young’s modulus of aged samples. There was a drastic reduction of modulus when a neat HDPE was aged, a noticeable ~26% reduction after 20 weeks of ageing. In contrast, FA/HDPE samples provided exceptional results with a lesser reduction in modulus over ageing times. Amongst aged FA/HDPE composites, FA15%/HDPE showed the least decrease of ~5% in Young’s modulus when aged for 20 weeks. This extraordinary result is attributed to the stabilising effect of fly ash fillers that adsorb light from the entire range of the solar spectrum and limit penetration of high-energy photons into the surrounding polymer matrix, eventually limiting polymer degradation. Figure 6B shows the effect of ageing on the tensile strength of HDPE and FA/HDPE samples. The tensile strength gradually decreases with increasing filler percentage respective to ageing duration. FA10%/HDPE composite showed the highest strength of ~29.3 MPa after 20 weeks of ageing (showing only ~5% reductions from the fresh sample) compared to other specimens. Considering the prominent Young’s modulus of 20 weeks aged FA15%/HDPE composite, it will also be noteworthy to evaluate the reduction of tensile strength. After 20 weeks of ageing, the FA15%/HDPE sample showed ~9% reduction in tensile strength. Combination of tensile modulus and strength is superior for FA15%/HDPE composite than other types. Compared to a previous ageing study on carbon nanotube reinforced HDPE composites, our FA/HDPE encountered lesser loss of tensile strength, which supports the greater significance of our current work. Overall, the filler concentration, their morphology, geometrical parameters and filler-matrix IFSS are the main causes of change in tensile properties of the aged composites.

## 4. Conclusions

Fly ash filled high density polyethylene (FA/HDPE) composites with three different weight fractions (5, 10 and 15%) of filler were prepared by melt processing and characterised for thermal, morphological and tensile properties. Thermogravimetric tests showed that the composites are of great uniformity in terms of filler distribution. Compared to neat HDPE polymer, incorporation of 15 wt% fly ash filled FA/HDPE composite sample showed a ~200% increase in Young’s modulus without the deterioration of tensile strength. The durability of prepared composites was studied by environmentally ageing (UV and moisture exposure) the samples, and the morphological and mechanical properties were evaluated accordingly. The utility of FA/HDPE composites for long-term usage was found more feasible compared to neat HDPE. While 10 wt% fly ash content was better suited for preserving tensile strength after 20 weeks of ageing, considering the combination of Young’s modulus and tensile strength, 15 wt% fly ash containing HDPE composite was found to be best performed. Compared to a freshly made composite, the later composite showed only ~5% decrease in Young’s modulus and ~9% decrease in Young’s modulus when aged for 20 weeks. The extraordinary ability of highly concentrated fly ash to adsorb high-energy photons from UV light restricted the photon-induced polymer matrix degradation, making the material suitable for long term outdoor usage.

## Data Availability

The data presented in this study are available on request from the corresponding author.
